# Ageing, multimorbidity and polypharmacy shape prosthodontic case-mix in undergraduate clinics: a 9-year retrospective cohort study of 1,205 patients in Germany

**DOI:** 10.1186/s12903-026-07977-5

**Published:** 2026-02-26

**Authors:** Anna-Lena Hillebrecht, Simon Maria Bliß, Kirstin Vach, Philipp Linde, Ralf J. Kohal, Kerstin Rabel, Daniel R. Reissmann, Benedikt C. Spies

**Affiliations:** 1 Department of Prosthetic Dentistry, Center for Dental Medicine, Faculty of Medicine, Medical Center ‑ University of Freiburg, University of Freiburg, Freiburg, Germany; 2 Institute of Medical Biometry and Statistics, Faculty of Medicine, Medical Center ‑ University of Freiburg, University of Freiburg, Freiburg, Germany; 3 Department of Radiation Oncology, Cyberknife and Radiation Therapy, Faculty of Medicine, University Hospital of Cologne, University of Cologne, Cologne, Germany; 4 Department of Prosthodontics and Materials Science, University Medical Center Leipzig, Leipzig, Germany

**Keywords:** prosthodontics, gerodontology, undergraduate education, dental education, geriatrics, multimorbidity, polypharmacy

## Abstract

**Background:**

Demographic ageing and increasing multimorbidity are transforming prosthodontic care, with growing demands related to medical complexity, polypharmacy, and long-term maintenance. It remains unclear to what extent these changing care realities are already reflected in undergraduate prosthodontic teaching clinics. This study aimed to characterize the age profile, systemic health, oral health, and prosthodontic treatment patterns of patients treated in an undergraduate prosthodontic clinic over nine years (2011–2019).

**Methods:**

This retrospective observational study included 1,205 consecutive patients treated in undergraduate prosthodontic courses at a German university dental center. Demographics, comorbidities, medication use, number of remaining teeth, Decayed-Missing-Filled-Teeth-(DMFT) Index and periodontal indices were extracted from electronic records. Prosthodontic treatment was categorized as fixed, removable, or combined (fixed–removable). Descriptive statistics, chi-square tests, and regression models were applied.

**Results:**

The cohort (45% women) had a mean age of 60.7 (± 12.3) years; mean age varied over the study period without a consistent linear trend. The most frequent comorbidities were cardiovascular diseases (42.3%), allergies (29.4%), and bleeding disorder/anticoagulation (19.1%). Polypharmacy (≥ 5 medications) increased from 6.8% (2011) to 18.6% (2019) and was more common at higher age. Patients had on average 16.8 (± 8.3) teeth at baseline and 14.5 (± 9.2) after treatment; baseline DMFT was 24.3 (± 6.8) and increased with age. Overall, 37.5% received fixed, 13.2% removable, and 49.3% combined prosthodontics. In multivariable analyses, higher age and higher baseline DMFT increased the odds of removable (vs. fixed) treatment, whereas more remaining teeth favored fixed treatment.

**Conclusions:**

Undergraduate prosthodontic clinics predominantly manage older, multimorbid, and often polymedicated patients. Treatment patterns are primarily explained by baseline oral and systemic conditions, underscoring the need for training in risk-adapted planning, medication-related risk management, and maintenance-oriented care.

**Supplementary Information:**

The online version contains supplementary material available at 10.1186/s12903-026-07977-5.

## Background

Like all medical disciplines, dentistry is increasingly confronted with older patients who often present with multimorbidity and functional limitations [1, 2]. On a global scale, the demographic shift is unprecedented: by 2050, the proportion of individuals aged 65 years and older will nearly double, reaching 16% of the world’s population [3]. Oral diseases in this age group rank among the most prevalent non-communicable conditions worldwide, with severe tooth loss alone affecting over 270 million people [4]. Tooth loss has been linked to adverse systemic and cognitive outcomes in older adults [5]. The functional sequelae, impaired mastication, with reduced nutrition and impaired quality of life, underscore the need for care models and training that anticipate complex prosthodontic decision-making and long-term maintenance [6, 7]. Oral diseases in ageing populations represent one of the most neglected public health challenges, requiring radical action to integrate oral health into general health agendas [6, 8]. The German situation is part of a broader challenge for dental education and oral care. Population projections anticipate that by 2060, more than one fifth of the German population will be over 67 years of age. Between 2017 and 2021, the number of people officially classified as in need of long-term care increased from 3.41 to nearly 5 million, with the highest growth observed among those aged 80 years and older [9]. This population exhibits poorer oral health than older adults without care needs: they have fewer remaining teeth, higher caries prevalence, and greater dependence on support for daily oral hygiene [10]. Data from the Fifth and Sixth German Oral Health Studies (DMS V/VI), the largest population-based oral health surveys in Germany, demonstrate that while oral health has improved overall, care-dependent older adults continue to exhibit particularly high preventive and therapeutic treatment needs that remain insufficiently addressed [11, 12]. Recent claims-data from Germany show that among the very old (≥ 75 years), utilization of prosthetic services declines with increasing age, underscoring structural barriers to maintaining prosthetic care at high age [13]. Epidemiological data highlight the urgent need to prioritize frail and care-dependent older adults. Yet national curriculum mapping indicates that German undergraduate programs only partially address this mandate: most schools disperse gerodontology content across general lectures, and only about half offer dedicated theoretical or practical instruction [14]. This gap aligns with concerns raised in European curriculum guidelines, which emphasize the necessity of structured and compulsory gerodontology training to adequately prepare students for the demographic realities of future practice [15]. To meet the growing treatment needs of an ageing and increasingly care-dependent population, undergraduate dental education must provide the foundational competencies for dental care and foster motivation for continuing education [16–18].

Despite increasing treatment needs in older and care-dependent patients, little is known about the actual case-mix represented in undergraduate prosthodontic training clinics. Given that prosthodontics is the discipline most directly confronted with the sequelae of tooth loss and functional decline, it provides a natural lens to examine whether current undergraduate training adequately reflects the case-mix of future practice. Providing prosthodontic care for older and care-dependent patients requires practical competencies such as assessing treatment capacity and oral function, adapting therapy to reduced resilience and oral hygiene ability, and planning and maintaining prosthetic solutions under complex conditions. These competencies must be developed through structured clinical training in undergraduate courses, in line with competency-based medical education frameworks that emphasize outcome-oriented acquisition of knowledge, skills, and professional attitude [16]. From a medical education perspective, it is essential to determine which age groups and patient structures are actually represented in undergraduate prosthodontic courses. Equally important is to assess whether these learning experiences prepare students for those populations with the highest treatment needs, and whether aspects such as reduced treatment capacity, limited adaptability, impaired oral-hygiene ability, and collaboration with caregivers are adequately addressed. Amid ongoing demographic change, the growing share of older and often multimorbid patients in undergraduate clinics poses new challenges for teaching, organization, and the financing of prosthodontic care [19]. This analysis corresponds to the first step of Kern’s six-step model of curriculum development, namely the systematic needs assessment that forms the basis for defining goals, educational strategies, and curricular integration [20]. A sustainable solution lies in designing curricula that systematically equip dental students with the competencies required to manage older and care-dependent patients. This necessitates structured, developmentally appropriate clinical experiences with such patient groups during training, ensuring that graduates are prepared to integrate age-associated complexities into prosthetic treatment planning in a responsible and safe manner.

Against this background, the present study aimed to describe the age distribution, number of teeth, caries experience, and types of prosthetic treatment among patients treated in undergraduate courses between 2011 and 2019.

## Methods

### Study design and setting

This retrospective, single-center observational study was conducted at the Department of Prosthetic Dentistry, University Medical Center Freiburg, Germany. The study was approved by the Ethics Committee of the University of Freiburg (EK Freiburg 22-1398-S1 retro). The requirement for informed consent was waived due to the retrospective analysis of anonymized routine clinical data. Clinical trial registration in the German Clinical Trials Register (DRKS00030542), registered on 28 August 2023. The study followed the STROBE guidelines for observational research.

The undergraduate prosthodontic clinic at the University Medical Center Freiburg provides prosthodontic care within scheduled teaching sessions under close specialist supervision. Patients are recruited via routine intake at the university dental center and referrals from other departments within the center (e.g., conservative dentistry/periodontology/oral surgery), as well as self-referrals. As typical for teaching settings, treatment planning and delivery are embedded in course structures with fixed treatment slots and increased chairside time compared with routine specialist care. Cost coverage follows the German statutory health insurance framework, with patient co-payments depending on the individual treatment plan; in our setting, the teaching environment may influence patients’ willingness to accept longer treatment pathways.

### Participants and inclusion criteria

All patient records from undergraduate prosthodontic courses between January 2011 and December 2019 were included. Patients were eligible for treatment in undergraduate prosthodontic courses provided they were able to participate in treatment planning and follow-up appointments. Adequate communication in German or English was required to ensure informed consent and safe clinical care. Exclusion criteria for treatment in the student courses included insufficient proficiency in German or English to allow effective communication, active infectious diseases, and markedly reduced treatment capacity due to severe systemic illness. These criteria reflect routine safety and organisational requirements of undergraduate clinical teaching.

Data were obtained from electronic patient records used for routine clinical documentation. Data beyond 2019 were excluded due to COVID-19–related disruptions. Records without prosthodontic treatment were excluded, resulting in 1,205 consecutive patients. Each year’s cohort consisted of different patients treated during that specific course period; patients were not followed longitudinally across years. Where data fields were unavailable, variables were coded as missing and retained in the dataset; no imputation was performed.

No sample size calculation was performed given the retrospective design.

### Data sources and variables

Patient information was extracted from patient records and included:

*Sociodemographics*: patient’s age, gender, type of health insurance.

Comorbidities were categorized into clinically meaningful groups based on the structure of the electronic patient records and established medical classifications. The following categories were used:

- Cardiovascular diseases (e.g., arterial hypertension, coronary heart disease)
- Pulmonary diseases (e.g., asthma, COPD)
- Neurological diseases (e.g., history of stroke, epilepsy, dementia)
- Metabolic diseases (e.g., diabetes mellitus)
- Psychiatric diseases (e.g., depression)
- Increased bleeding tendency (e.g., anticoagulation)
- Infectious diseases (e.g., HIV, HAV/HBV)
- Sensory organ diseases (e.g., cataract, glaucoma)
- Cancer (e.g., malignant neoplasms)
- Gastrointestinal diseases (e.g., Crohn’s disease)
- Thyroid diseases (e.g., hyper-/hypothyroidism)
- Allergies (e.g., medications)
- Musculoskeletal diseases/mobility limitations (e.g., arthrosis, osteoporosis)

A comorbidity score was calculated for each patient as the sum of the categories present (theoretical range 0–13), with higher values indicating a greater overall medical burden. In accordance with common definitions of multimorbidity, the presence of two or more comorbid conditions (score ≥ 2) was classified as multimorbidity [21].

*Medication count*: total number of regularly taken systemic medications at baseline (as recorded).

*Dental status*: number of teeth present, DMFT and components (DT, MT, FT) at baseline and after treatment; periodontal indices (when available).

### Prosthodontic treatment types

Prosthodontic treatment categories were coded as follows:

*Fixed dental prostheses*: crown; bridge; implant-supported crown or bridge; resin-bonded bridges.

*Removable dental prostheses*: model-cast partial denture; complete denture

Combined dental prostheses: Cases were classified as combined prosthodontics when both fixed and removable components were part of the same prosthodontic treatment concept. This category reflects a common treatment approach in the German statutory health insurance system, where fixed restorations (e.g. crowns or bridges) are frequently combined with cast metal removable partial dentures to achieve functionally and economically viable solutions. Combined prosthodontics included, but were not limited to, telescopic dentures, attachment-retained removable partial dentures, implant-supported overdentures, bar-retained dentures, and cover dentures.

Cases with fixed restorations only (e.g. crowns, fixed bridges, implant-supported fixed prostheses) were classified as fixed prosthodontics, while cases involving exclusively removable restorations without fixed abutments (e.g. complete dentures or conventional removable partial dentures without fixed components) were classified as removable prosthodontics.

*Materials for crowns and bridges*: ceramic, non-precious metal (NEM), precious metal (EM), titanium, metal-ceramic. A “material unit” was counted per patient as the number of respective material components in the final prosthetic reconstruction.

*Patient age categories*: <65, 65–75, and >75 years.

### Educational framing

In addition to clinical descriptors, the study was conceived as Step 1 (“problem identification and general needs assessment”) within the Kern curriculum development framework. Findings were prospectively earmarked to inform learning objectives, case allocation, and assessment formats (e.g., structured oral examinations) related to care of older, multimorbid patients.

### Statistical analysis

Descriptive statistics summarized variables with mean values and standard deviations or relative and absolute frequencies. For comparisons of categorical variables Chi-square-tests were used. A linear regression model was used to analyse the influence of several covariates on the comorbidity score. With the help of logistic regression models the association between comorbidities and age and gender as well as the association between treatment type (fixed vs. removable) and predictors (age, number of teeth, DMFT) were analysed. Negative binomial regression models adjusting for temporal trends were used to analyse the influence of several covariates on the number of medications. All analyses took into account the clustering of data, where indicated. Analyses were conducted using Stata (version 19, StataCorp, College Station, TX, USA). The level of statistical significance was set at α < 5%.

## Results

### Study population

A total of 1,205 patients were included with 45% women (*n* = 543) and 55% men (*n* = 662). Statutory health insurance covered 96.9% (*n* = 1,168) of the patients, whereas private insurance covered 3.1% (*n* = 37).

Mean patient age varied between 56.4 and 63.6 years over the study period without a consistent linear trend over time (Table 1).

**Table 1:** Demographic data by year (2011–2019)

Year	Age in years		*n* (f/m)
	Mean value	Standard deviation	
2011	63.6	10.1	44 (23/21)
2012	58.3	13.0	138 (60/78)
2013	58.6	12.0	137 (55/82)
2014	56.4	12.2	126 (63/63)
2015	58.1	12.0	133 (61/72)
2016	61.5	12.6	133 (59/74)
2017	63.5	12.1	164 (79/85)
2018	60.1	11.9	163 (76/87)
2019	62.0	12.8	167 (67/100)
Overall	60.7	12.3	1205 (543/662)

*f* Female, *m* male

### Comorbidities and polypharmacy

The mean comorbidity score was 2.1 ± 1.4 (observed range 0–9), and 64.7% of patients met the criterion for multimorbidity (≥ 2 comorbidities).

Cardiovascular disease was the most prevalent comorbidity (42.3%), followed by allergies (29.4%), metabolic disease (18.5%), thyroid disease (18.0%), and bleeding disorders or anticoagulation therapy (19.1%). Sensory organ diseases affected 13.9% of participants, while pulmonary, gastrointestinal, and neurological disorders each occurred in approximately 11–13% of the sample. Psychiatric diseases were least frequent (2.1%).

**Fig. 1:**
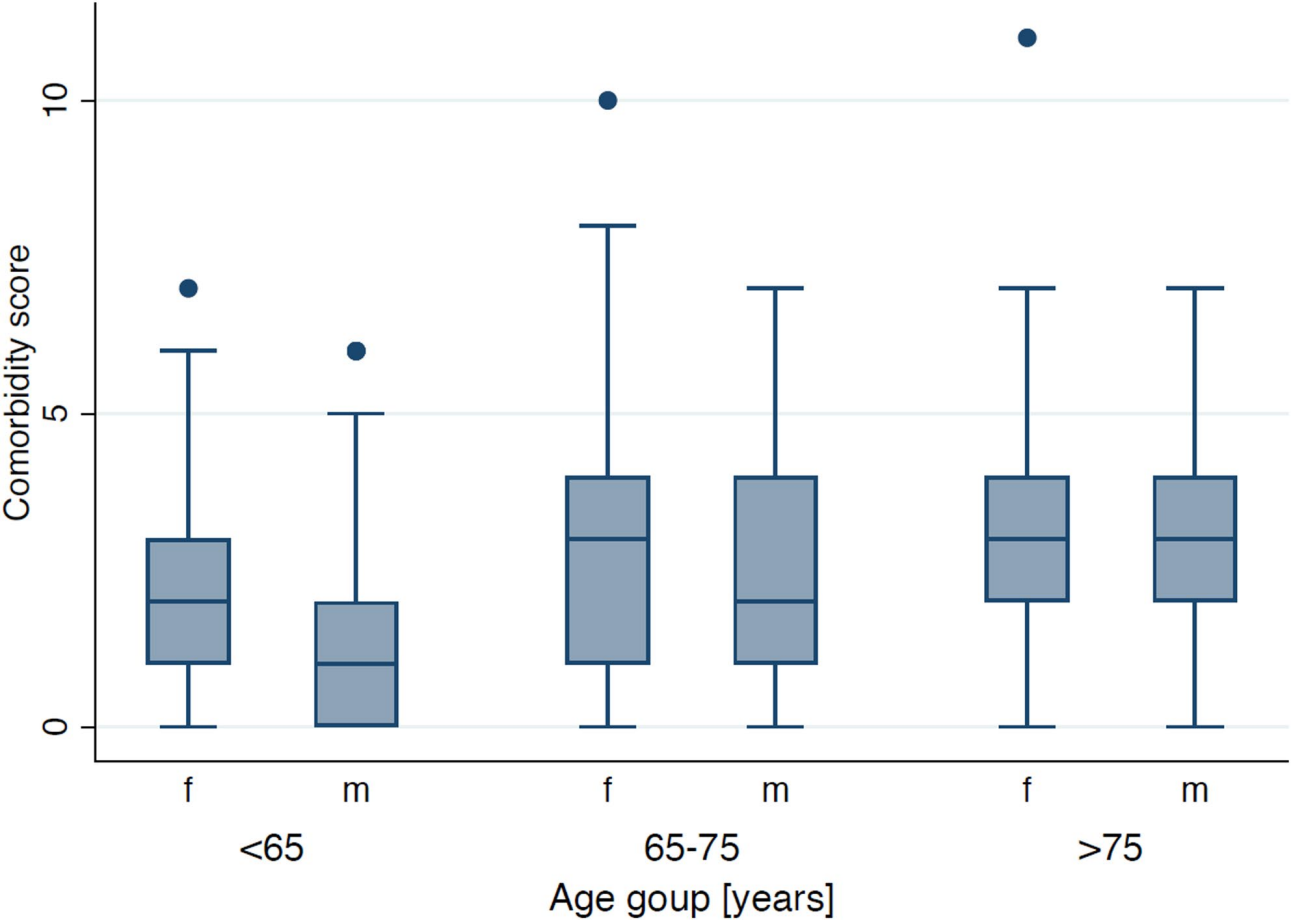
Comorbidity score by age group and gender. Box–whisker plots show the distribution of summed comorbidity categories among patients treated in undergraduate prosthodontic courses, stratified by age (< 65, 65–75, > 75 years) and gender (f, m). Median comorbidity scores increased with age and remained stable between the older age groups

Increasing age was significantly associated with higher odds of several comorbidities, including cardiovascular disease, metabolic disease, bleeding disorders/anticoagulation, sensory organ disease, thyroid disease, and mobility limitation/musculoskeletal disease (all *p* < 0.05). The strongest age-related effect was observed for sensory organ disease (OR 1.10 per year, 95% CI 1.08–1.12) (Table 2; Figure 1).

**Table 2:** Prevalence of comorbidities and associations with age and gender (n = number of patients). OR = Odds ratio. Yr = year. Significant effects (*p* < 0.05) are shown in bold. Total *N* = 1,205

Comorbidity	n (%)	Age OR/yr (95% CI), *p*-value	Male vs. Female OR (95% CI), *p*-value	Year since 2011 OR (95% CI), *p*-value
Cardiovascular disease	509 (42.3)	1.08 (1.07–1.09), **< 0.001**	0.89 (0.68–1.17), 0.410	1.04 (0.98–1.09), 0.180
Pulmonary disease	136 (11.3)	1.02 (1.01–1.04), **0.010**	0.71 (0.48–1.05), 0.080	1.00 (0.93–1.08), 0.950
Neurological disorder	156 (12.9)	1.01 (0.99–1.02), 0.450	0.94 (0.65–1.36), 0.740	1.15 (1.07–1.24), **< 0.001**
Metabolic disease	223 (18.5)	1.04 (1.02–1.05), **< 0.001**	1.18 (0.85–1.64), 0.320	1.06 (1.00–1.13), 0.050
Bleeding disorder / Anticoagulation	230 (19.1)	1.07 (1.05–1.09), **< 0.001**	1.96 (1.39–2.77), **< 0.001**	1.10 (1.04–1.18), **< 0.001**
Infections	143 (11.9)	1.03 (1.00–1.06), 0.080	1.00 (0.54–1.85), 0.990	0.98 (0.88–1.10), 0.760
Sensory organ disease	167 (13.9)	1.10 (1.08–1.12), **< 0.001**	1.17 (0.81–1.69), 0.410	1.09 (1.01–1.18), **0.020**
Cancer	127 (10.5)	1.05 (1.03–1.07), **< 0.001**	0.95 (0.63–1.44), 0.810	1.03 (0.95–1.12), 0.470
Gastrointestinal disorder	143 (11.9)	1.02 (1.00–1.03), **0.010**	1.14 (0.77–1.68), 0.510	0.96 (0.89–1.04), 0.330
Thyroid disease	217 (18.0)	1.03 (1.02–1.05), **< 0.001**	0.19 (0.13–0.27), **< 0.001**	1.07 (1.00–1.14), **0.040**
Allergies	354 (29.4)	0.99 (0.98–1.00), 0.050	0.37 (0.28–0.50), **< 0.001**	0.94 (0.89–0.99), **0.020**
Mobility limitation / Musculoskeletal disease	172 (14.3)	1.04 (1.02–1.05), **< 0.001**	0.75 (0.53–1.06), 0.100	1.02 (0.96–1.09), 0.540
Psychiatric disease	25 (2.1)	0.98 (0.95–1.01), 0.190	1.05 (0.41–2.65), 0.920	1.06 (0.91–1.24), 0.440

Bold type indicates statistically significant associations (*p* < 0.05)

Only two comorbidities showed significant sex differences: males had higher odds of bleeding disorders/anticoagulation (OR 1.96, 95% CI 1.39–2.77, *p* < 0.001), whereas females had significantly higher odds of thyroid disease (OR 0.19, 95% CI 0.13–0.27, *p* < 0.001). No significant sex effects were observed for cardiovascular, metabolic, or neurological diseases.

There was a significant temporal trend for several comorbidities recorded since 2011. Sensory organ disease (OR 1.09 per year, *p* = 0.020) and bleeding disorders/anticoagulation (OR 1.10 per year, *p* < 0.001) increased over time, whereas allergies slightly decreased (OR 0.94 per year, *p* = 0.020). No meaningful changes over time were noted for cardiovascular or pulmonary disease.

**Fig. 2:**
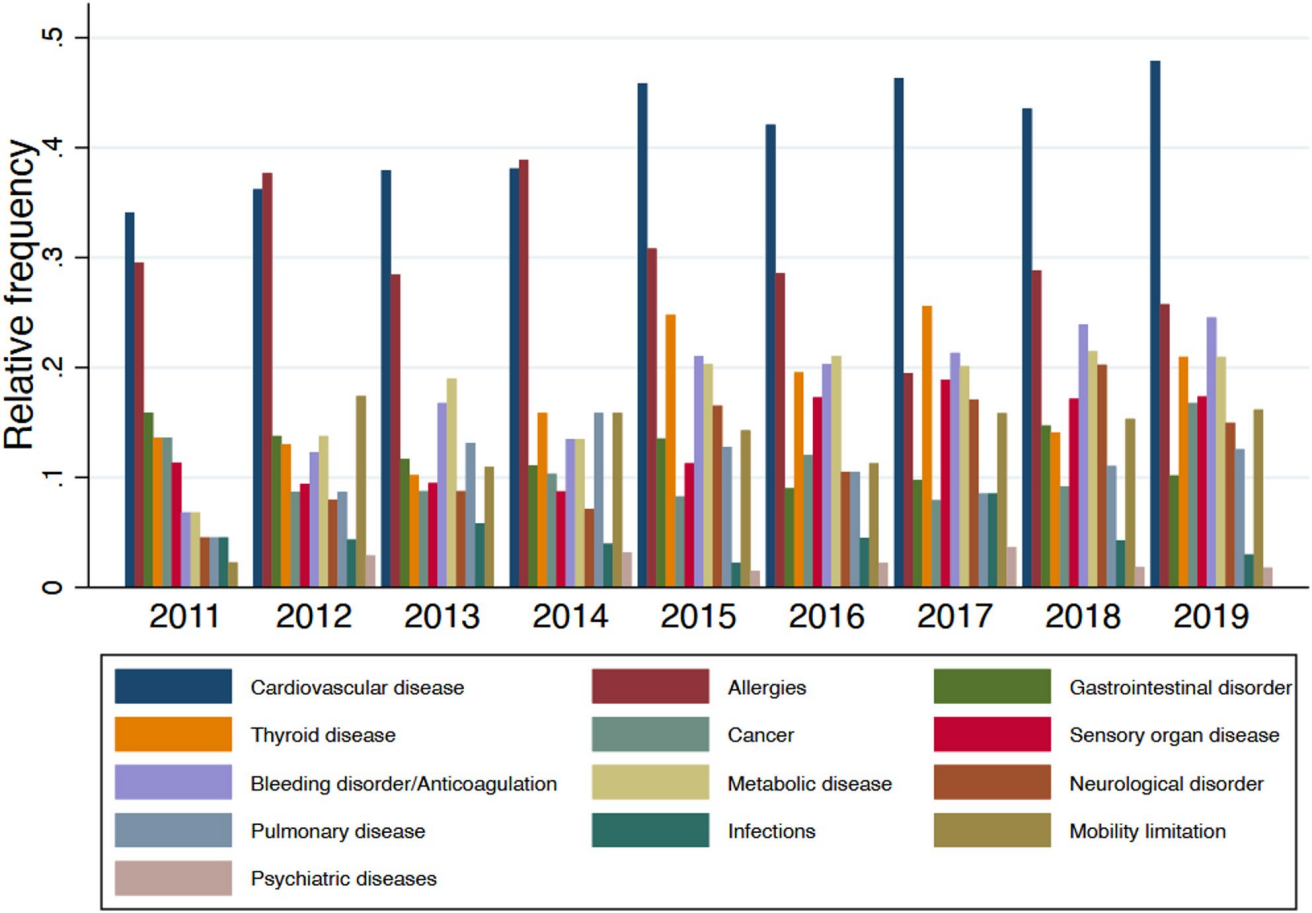
Relative frequencies of comorbidities among patients treated in undergraduate prosthodontic courses (2011–2019)

Over time, several comorbidities became more frequent: bleeding disorders/anticoagulation (OR 1.10 per year, *p* < 0.001), neurological disorders (OR 1.15, *p* < 0.001), sensory organ disease (OR 1.09, *p* = 0.020), and thyroid disease (OR 1.07, *p* = 0.040). Metabolic disease showed a borderline increase (OR 1.06, *p* = 0.050), whereas allergies decreased slightly (OR 0.94, *p* = 0.020). In a time-adjusted model, comorbidity scores increased with age (β = 0.05 per year, *p* < 0.001) and were lower in women (β= − 0.37, *p* < 0.001). The relative frequencies of individual comorbidities across the cohort are summarized in Figure 2.

**Fig. 3:**
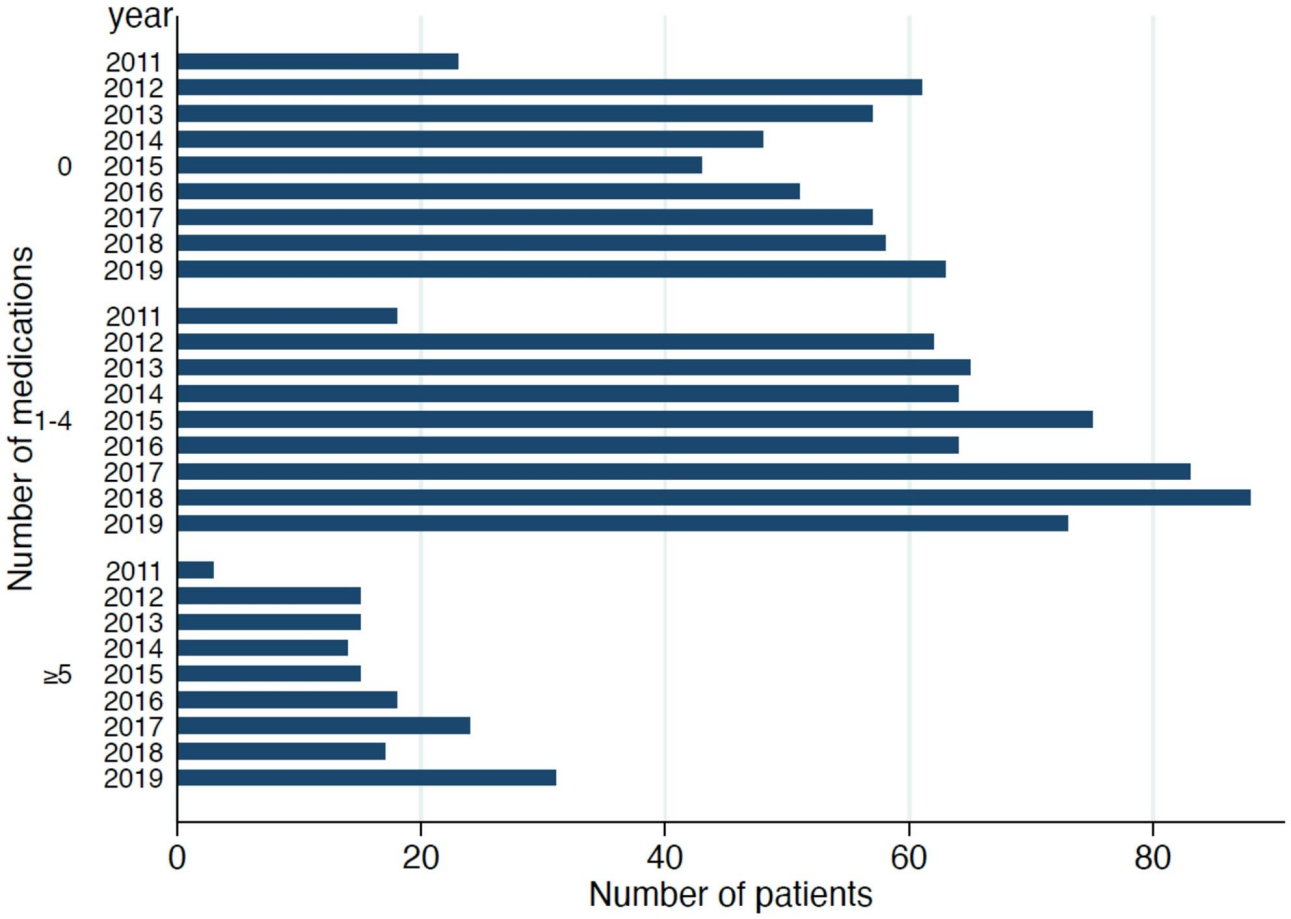
Distribution of polypharmacy categories across the study period (2011–2019). Patients were stratified into three groups: no medication (0), low medication burden (1–4 medications), and polypharmacy (≥ 5 medications)

Over time, polypharmacy (≥ 5 medications) rose from 6.8% in 2011 to 18.6% in 2019, while 1–4 medications remained the most frequent category (2011: 40.9%; 2019: 43.7%). Overall, 38.3% of patients reported no regular medication, 49.1% used 1–4 drugs, and 12.6% fulfilled the criterion for polypharmacy. Polypharmacy increased with age (< 65: 7.6%; 65–75: 19.0%; >75: 23.5%; *p* < 0.001). Figure 3 shows the temporal distribution of polypharmacy using absolute patient numbers to illustrate changes in case volume, while percentages are reported in the text for comparability across cohorts.

### Oral health status

**Fig. 4:**
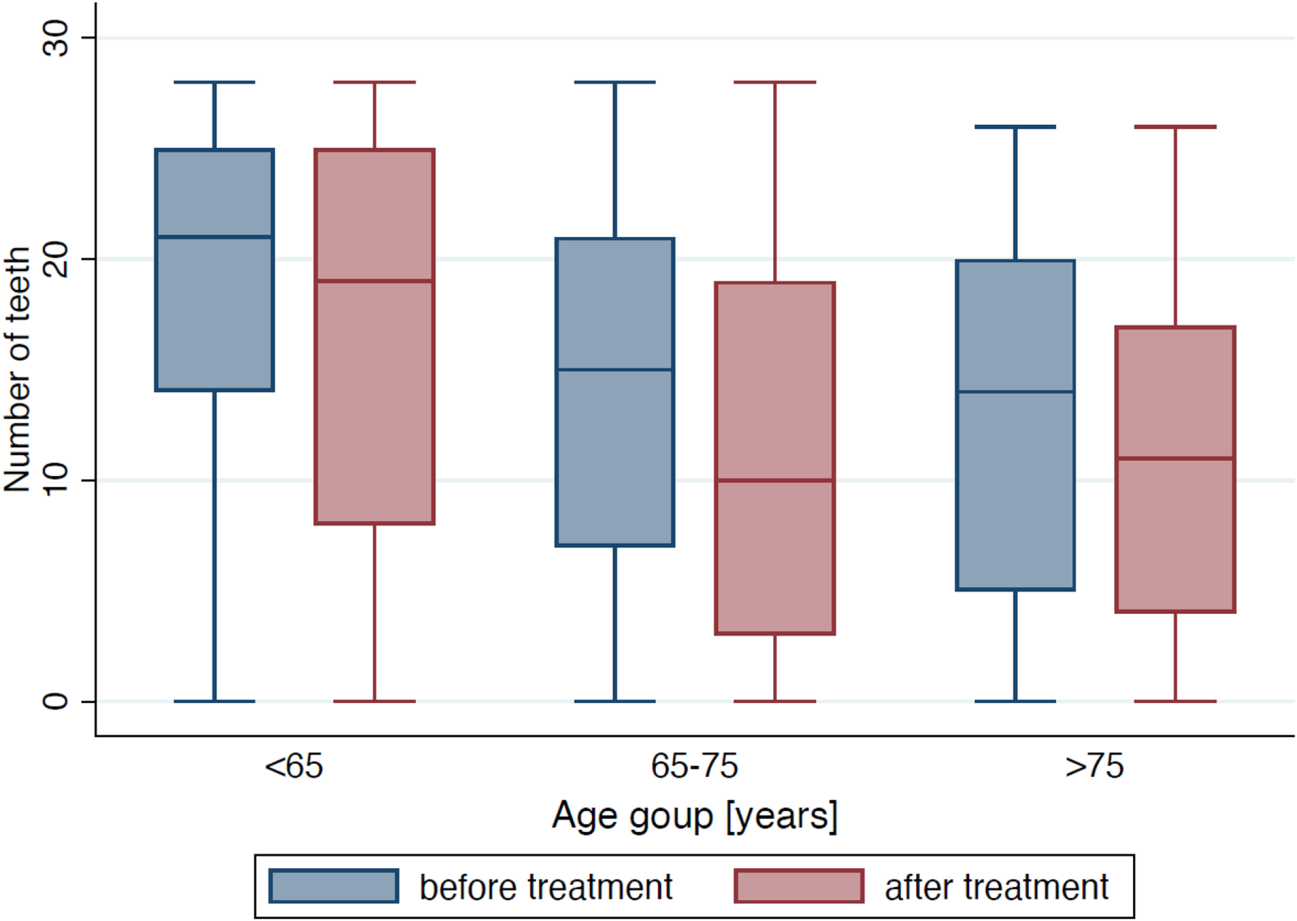
Number of teeth before and after treatment by age group. Box–whisker plots show the number of remaining teeth at baseline (blue) and after treatment (red), stratified by age (< 65, 65–75, > 75 years)

At baseline, patients presented with a reduced but heterogeneous dentition, with the number of remaining teeth decreasing with age and further declining slightly after treatment; this pattern is illustrated in Figure 4. Baseline mean DMFT was 24.3 ± 6.8, with mean values of 23.2 ± 7.2 (< 65 years), 25.6 ± 6.0 (65–75 years), and 26.6 ± 5.3 (> 75 years). Periodontal indices were available for 338 patients and decreased from 29.8% to 17.6% after treatment, with no significant differences between age groups. The mean Periodontal Screening Index (PSI) sextant value (*n* = 264) was 1.5 ± 1.3 with an average sum of 2.0 ± 5.2. Implant therapy was rare, and implant status remained unchanged in more than 90% of patients.

### Associations between age, medication use, and dental outcomes

A negative binomial regression model adjusted for temporal trends showed a significant association between age and number of medications (β = 0.04 per year, *p* < 0.001), whereas gender and baseline DMFT were not significant predictors (*p* = 0.100 and *p* = 0.676, respectively).

**Fig. 5:**
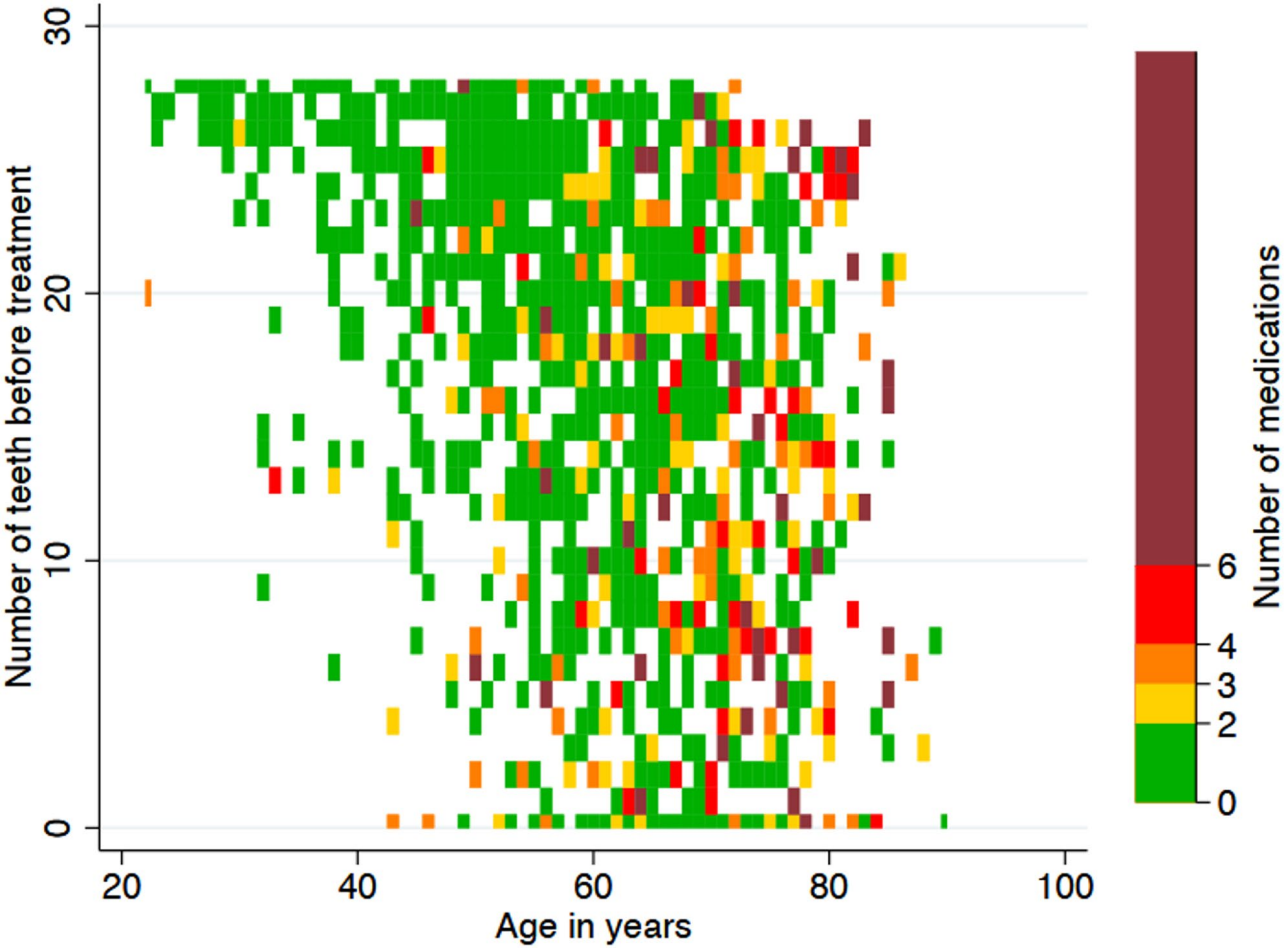
Relationship between age, number of teeth before treatment, and polypharmacy. The heatmap displays the joint distribution of age, medication counts, and baseline teeth

In linear models adjusted for age and gender, each additional medication was associated with fewer post-treatment teeth (β = − 0.214, *p* = 0.035), whereas age showed a stronger negative association (β = − 0.272 per year, *p* < 0.001). For post-treatment DMFT, age remained the dominant predictor (β = +0.155 per year, *p* < 0.001), while the number of medications showed no independent association (β = +0.067, *p* = 0.269). Figure 5 illustrates the distribution of age, medication counts, and baseline teeth. The corresponding distribution for post-treatment dentition and medication burden is shown in Supplementary Figure S2.

### Prosthodontic treatment patterns

**Fig. 6:**
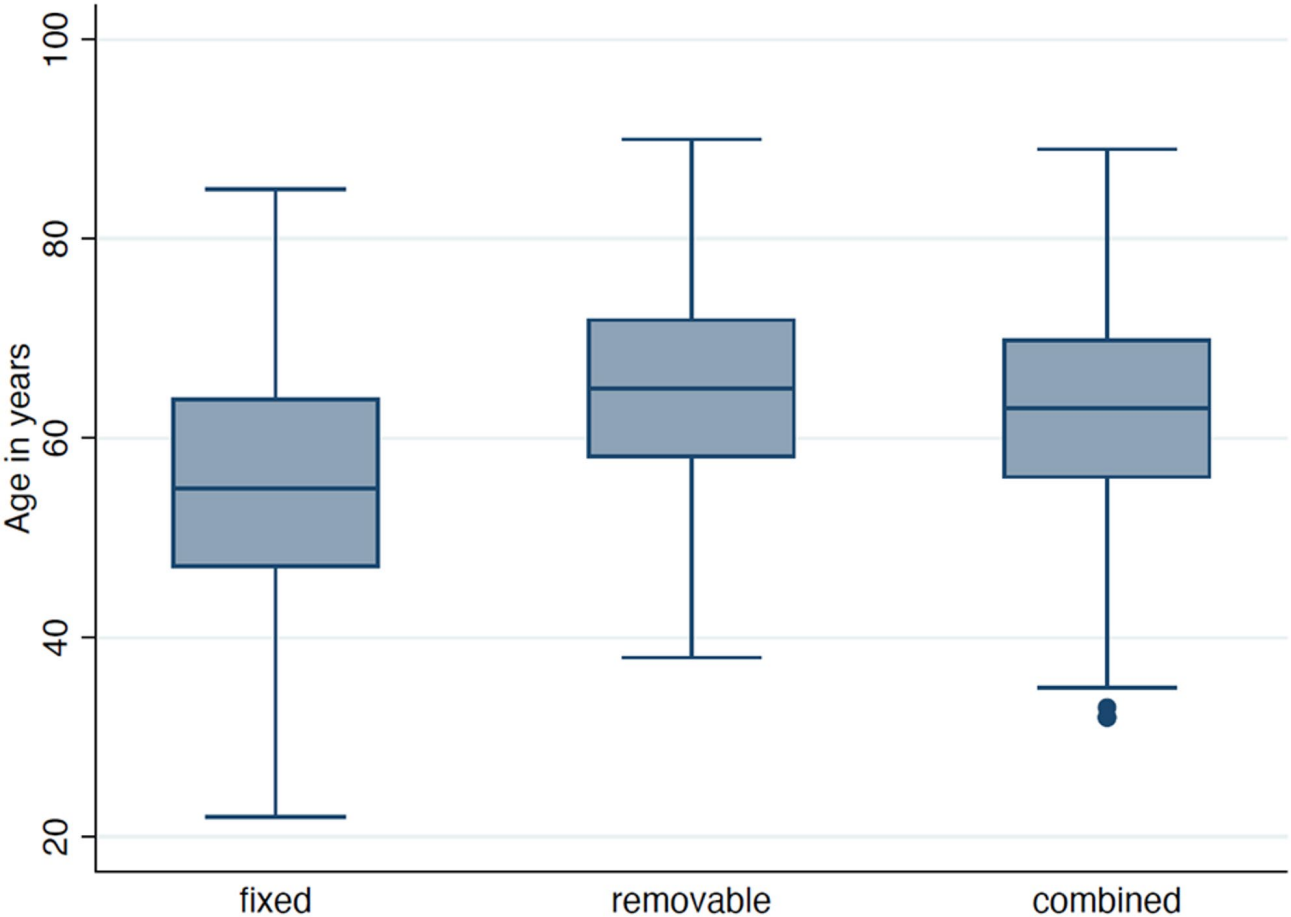
Distribution of patient age across prosthodontic treatment categories (fixed, removable, combined)

In the overall cohort, 49.3% of cases involved combined prosthodontic treatment (fixed + removable), 37.5% were fixed, and 13.2% were removable restorations. The proportion of combined prosthodontics increased with age, accounting for 43.8% of treatments in patients under 65 years, 58.9% in those aged 65–75 years, and 55.7% over 75 years (Fig. 6). Treatment distributions differed by gender (*p* = 0.024). Chi-square-tests confirmed differences in treatment distribution across age (*p* < 0.001), polypharmacy (*p* < 0.001), and gender (*p* = 0.024). Patients with polypharmacy (≥ 5 medications) were less often treated with fixed prosthodontics (9.3% fixed among polypharmacy vs. 48.3% fixed among 0 medication), and received more often combined/removable solutions. The distribution of age and pre-treatment dentition across fixed, removable, and combined prosthodontics, stratified by anticoagulation status, is illustrated in Supplementary Figure S1. Summing up, material units per patient across all reconstructions, non-precious metal (NEM) frameworks were most frequent (mean 1.92 units/patient; 2,308 units in 1,205 cases), followed by ceramic (0.85; 1,023) and metal-ceramic (0.50; 601). Precious metal (EM) was rare (0.17; 201), and titanium components were used infrequently.

### Predictors of treatment type

In exploratory logistic regression (*n* = 611), higher age and greater baseline DMFT were associated with an increased likelihood of receiving removable rather than fixed prosthodontic treatment, whereas a higher number of remaining teeth reduced this likelihood (all *p* < 0.001) (Table 3).

**Table 3:** Logistic regression analysis of predictors for receiving removable vs. fixed prosthodontics (*n* = 611). OR = odds ratio; CI = confidence interval

Predictor	OR	95% CI	*p*-value
Age (year)	1.07	1.05–1.09	< 0.001
DMFT	1.40	1.31–1.49	< 0.001
Remaining teeth (per tooth)	0.63	0.58–0.69	< 0.001

## Discussion

The patient population treated in undergraduate prosthodontic clinics is characterized by older age and substantial medical complexity. Mean patient age varied across study years without a statistically significant temporal trend. In contrast, the prevalence of several systemic conditions (e.g., thyroid disease, bleeding disorders/anticoagulation, sensory organ and neurological disorders) increased over time. As expected, the comorbidity score rose with age, and polypharmacy, defined as the use of five or more medications, almost tripled between 2011 and 2019. Together, these findings illustrate that multimorbidity and medication burden represent central characteristics of the prosthodontic case-mix encountered in undergraduate clinics.

Prosthodontic treatment patterns were primarily associated with baseline oral and systemic conditions rather than age per se. In multivariable analyses, a higher cumulative disease experience (DMFT) and fewer remaining teeth were the strongest determinants of removable versus fixed treatment, while age acted mainly as a surrogate marker for these underlying clinical characteristics. Specifically, each additional year of age increased the odds of removable treatment by 7%, each additional DMFT unit by 40%, whereas each remaining tooth reduced the odds by 37%. Accordingly, patients presenting with more compromised but still partially retained dentitions were more likely to receive combined fixed–removable solutions.

Nearly half of all patients (49.3%) were treated with combined prosthodontics. The high proportion of combined prosthodontic treatments reflects typical treatment strategies within the German statutory health insurance system, where combined fixed–removable solutions are frequently chosen for economic and functional reasons. This context should be considered when transferring the absolute distribution of treatment types to healthcare systems with different reimbursement structures. However, the observed association between increasing age, medical complexity and the need for more complex prosthodontic solutions is likely transferable across healthcare systems.

The dataset reflects a typical prosthodontic patient population, where teeth of limited prosthetic value are often extracted to enable strategic treatment planning and stable long-term outcomes [22].

Consistent with national and international data, prosthodontic care in ageing populations is increasingly shaped by medical complexity rather than chronological age alone. German insurance-based analyses have shown that among adults older than 75 years, utilization of prosthetic services declines despite increasing treatment needs [13], suggesting that older patients who still reach dental services represent a particularly demanding clinical subgroup. Similar patterns have been reported in Swiss and UK teaching clinics, where patient cohorts showed advanced tooth loss, extensive restorative histories, and increasing multimorbidity [23, 24]. Reported mean ages and multimorbidity prevalences in European undergraduate prosthodontic clinics closely match those observed in the present cohort [19, 23].

Compared with national epidemiological reference data (DMS V/VI), patients in this cohort exhibited higher DMFT scores and fewer remaining teeth, underscoring that undergraduate prosthodontic courses increasingly manage patients with functionally and prosthodontically high-risk profiles. Taken together, these findings indicate that undergraduate prosthodontic clinics are no longer limited to uncomplicated cases but increasingly reflect the demographic and epidemiological realities of contemporary dental practice.

### Educational implications

From a medical education perspective, the present analysis provides a structured needs assessment in line with Kern’s six-step model of curriculum development [20]. The findings demonstrate that student courses already expose learners to a prosthodontic patient population characterized by multimorbidity, reduced dentition, and complex treatment needs, features that reflect typical decision-making scenarios in prosthodontics [22]. Over the study period, comorbidities increased consistently, particularly bleeding disorders/anticoagulation, neurological disorders, sensory organ disease and thyroid disease. This indicates that undergraduate students were increasingly exposed to medically complex and multimorbid patients over time. However, the highest-need group, frail and care-dependent older adults, remains underrepresented; this is in line with observations from gerodontology education surveys [14]. At the same time, those patients who do present in student courses already show extensive and complex prosthodontic findings. Managing such treatment needs within the limited number of course days available to students is often not feasible [19]. Because the frailest, highly care-dependent individuals are generally excluded from student clinics for safety, ethical, and structural reasons and the patients who are treated already present age-associated needs, complementary educational formats are required.

### Strengths and limitations

Strengths of this study include the large, consecutive sample treated over nine years, detailed documentation of prosthodontic treatment patterns, and multivariable analyses.

Limitations include the retrospective, single-center design and the fact that undergraduate clinics do not treat the most frail or medically unstable patients, likely underestimating the full complexity of prosthodontic care in very old and care-dependent populations. Formal examiner calibration was not performed; however, key data were collected using standardized anamnesis forms and structured treatment planning, with all entries reviewed under specialist supervision. Observed treatment patterns may also partly reflect course structures, as only prosthodontic courses were included. We did not systematically assess financial incentives or co-payment differences, which may influence attendance and case-mix in teaching clinics.

## Conclusions

Undergraduate prosthodontic clinics predominantly treat older and multimorbid patients with a relevant and increasing burden of polypharmacy. Patients typically present with reduced but heterogeneous dentitions and high cumulative disease experience, resulting in complex prosthodontic treatment needs. Age, systemic health status, and the number and condition of remaining teeth were key determinants of treatment patterns, indicating a growing need for risk-adapted planning, preventive measures, and long-term maintenance strategies in undergraduate prosthodontic education.

## Supplementary Information

Supplementary Material 1.

## Data Availability

The datasets analysed during the current study are available from the corresponding author on reasonable request.

## Abbreviations

COPD: Chronic obstructive pulmonary disease
DMS: German Oral Health Study (Deutsche Mundgesundheitsstudie)
DMFT: Decayed, Missing and Filled Teeth
DT: Decayed Teeth
EM: Precious metal alloy (Edelmetall)
FT: Filled Teeth
HAV: Hepatitis A virus
HBV: Hepatitis B virus
HIV: Human immunodeficiency virus
NEM: Non–precious metal alloy (Nichtedelmetall)
OR: Odds ratio
PSI: Periodontal Screening Index
