# Supplementary material for: Ageing, multimorbidity and polypharmacy shape prosthodontic case-mix in undergraduate clinics: a 9-year retrospective cohort study of 1,205 patients in Germany
Source: BMC Oral Health. 2026 Feb 26;26:423. doi: 10.1186/s12903-026-07977-5 (PMC12955261; doi:10.1186/s12903-026-07977-5)
Supplement: Supplementary file 1 — Supplementary Material 1. [file 12903_2026_7977_MOESM1_ESM.docx]

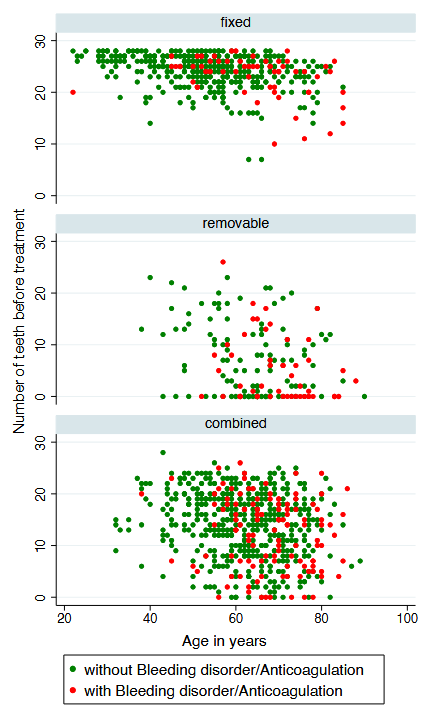


**Supplementary Figure S1**. Distribution of age and number of teeth before treatment in patients receiving fixed, removable, and combined prosthodontics, stratified by anticoagulation status. Red dots represent anticoagulated patients; green dots represent non-anticoagulated patients. Anticoagulated patients were present across all treatment categories and spanned a wide age range, underlining the complexity of the medical profiles encountered in undergraduate prosthodontic clinics.


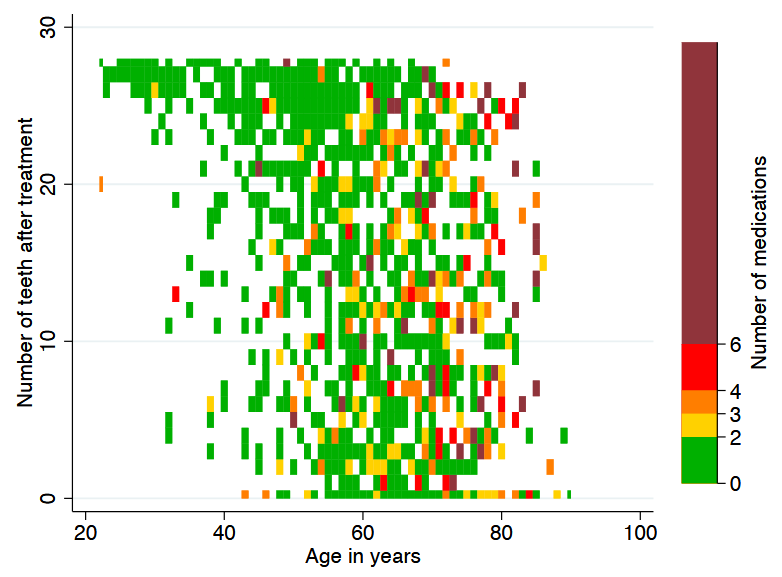


**Supplementary Figure S2.** Relationship between age, post-treatment dentition, and medication burden (n = 1,205). The y-axis shows the number of teeth after treatment; the x-axis shows age (years). Colors code the number of regularly taken medications (green = none; yellow to dark red colors = more medications). With increasing age, higher medication counts and lower tooth numbers become more frequent, while substantial interindividual variation remains—some older patients retain many teeth even with higher medication counts.
